# Nondestructive Detection for Egg Freshness Based on Hyperspectral Scattering Image Combined with Ensemble Learning

**DOI:** 10.3390/s20195484

**Published:** 2020-09-25

**Authors:** Dejian Dai, Tao Jiang, Wei Lu, Xuan Shen, Rui Xiu, Jingwei Zhang

**Affiliations:** 1 College of Artificial Intelligence, Nanjing Agricultural University, Nanjing 210031, China; djdai@njau.edu.cn (D.D.); 32217208@njau.edu.cn (T.J.); 32217320@njau.edu.cn (X.S.); 32217325@njau.edu.cn (R.X.); 2018812089@njau.edu.cn (J.Z.); 2 Jiangsu Province Engineering Laboratory of Modern Facility Agriculture Technology and Equipment, Nanjing 210031, China

**Keywords:** egg freshness, hyperspectral detection, hyperspectral scattering imaging, ensemble learning

## Abstract

Scattering hyperspectral technology is a nondestructive testing method with many advantages. Here, we propose a method to improve the accuracy of egg freshness, research the influence of incident angles of light source on the accuracy, and explain its mechanism. A variety of weak classifiers classify eggs based on the spectra after preprocessing and feature wavelength extraction to obtain three classifiers with the highest accuracy. The three classifiers are used as metamodels of stacking ensemble learning to improve the highest accuracy from 96.25% to 100%. Moreover, the highest accuracy of scattering, reflection, transmission, and mixed hyperspectral of eggs are 100.00%, 88.75%, 95.00%, and 96.25%, respectively, indicating that the scattering hyperspectral for egg freshness detection is better than that of the others. In addition, the accuracy is inversely proportional to the angle of incidence, i.e., the smaller the incident angle, the camera collects a larger proportion of scattering light, which contains more biochemical parameters of an egg than that of reflection and transmission. These results are very important for improving the accuracy of non-destructive testing and for selecting the incident angle of a light source, and they have potential applications for online non-destructive testing.

## 1. Introduction

The freshness of eggs is related to their nutritional value. It is the most concerned index of processing companies and consumers, and an important index in transportation and processing [1]. It can be detected using traditional biochemical methods, but they are destructive, time-consuming, and inefficient. Therefore, non-destructive testing technology has significant advantages in the detection of egg freshness and has attracted wide attention. Currently, egg freshness is tested using non-destructive techniques of spectral analysis [2,3], dielectric property [4,5], electronic nose [6,7], machine vision [8,9], and hyperspectral testing [10,11,12]. Especially, machine vision method was established for egg freshness with an R (correlation coefficient) value of 0.8653 [8]. The prediction model was established using near-infrared spectroscopy with an R value of 0.879 [13]. The freshness model was established by testing the volatile concentration of eggs by electronic nose with a low efficiency, thus, it is not suitable for the dynamic testing of production line [7]. The egg freshness was tested using reflectance near-infrared hyperspectral with an R value of 0.879 [10], which could achieve rapid and non-destructive classification of egg freshness. However, the model precision could not be further improved due to the great influence of eggshell colors.

Hyperspectral imaging technology is an emerging non-destructive testing technology that can obtain a large number of spatial image information samples of the frequency band and the spectral information of each pixel [14]. Currently, hyperspectral technology can be applied to non-destructive testing of the phenotype of grains, fruits, and vegetables [15,16,17]. However, there are only a few papers using it to study the freshness of eggs. Among them, using reflectance hyperspectrum to detect egg freshness can achieve fast and non-destructive grading of egg freshness with a correlation coefficient of 0.93 [18]. In addition, by using the optimal classification model (IRIV GA-SVM), the classification accuracy on the training set and test set achieved 99.25% and 97.87% respectively [19]. However, there are few studies on the non-destructive freshness detection of scattering and transmission hyperspectral. Meanwhile, the optical fiber scattering spectra have been used to study the surface and internal defects of apples and tomatoes with high accuracy [20], which indicated that it would be feasible to detect the freshness of eggs.

Herein, we proposed a method to improve the accuracy of egg freshness based on hyperspectral scattering imaging, and we researched the influence of incident angles on the accuracy and explained its mechanism. We found that stacking ensemble learning could be used to improve the highest accuracy of egg freshness, and the accuracy is inversely proportional to the incident angle. These results are useful for improving the accuracy of a classifier, important for selecting the incident angle of a light source with high accuracy, and they have potential applications in online non-destructive testing.

## 2. Materials and Methods

### 2.1. Experimental Materials

A total of 350 eggs (pink shell, mass 31.5–46.6 g, equatorial diameter 32.8–41.9 mm) were purchased from Panchu Mechanized Chicken Farm, Nanjing, Jiangsu Province, China. They were all produced on the day of purchase and stored at room temperature after cleaning. These eggs were divided into two groups, i.e., the data group and the calibration group, with 200 and 150 eggs, respectively. The data group was used to collect hyperspectral images, and the calibration group was used to measure the Haugh unit.

### 2.2. Hyperspectral Imaging System

The hyperspectral instrument was a GaiaSorter-Dual “Gaia” dual-camera all-band hyperspectral sorter. Its main components include a uniform light source, a dual spectrum camera, an electronic control transfer module, a computer with a control software, etc. The dual spectrum camera included two hyperspectral cameras, Camera 1 (Image-λ-V10E, wavelength range 391.6–1044.1 nm, and resolution 2.5 nm) and Camera 2 (Image-λ-N25E, wavelength range 1044.1–2528.1 nm, and resolution 5.6 nm).

**Figure 1:**
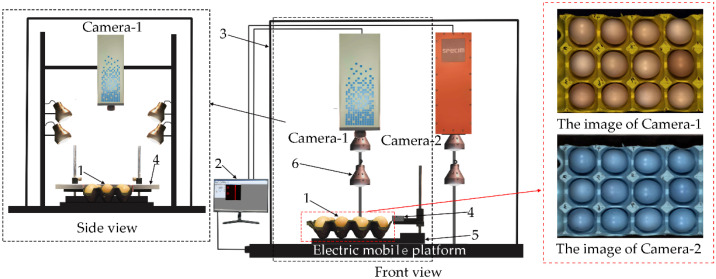
Reflection hyperspectral imaging system. (**1**) Pink-shell egg; (**2**) Computer; (**3**) Black box; (**4**) Calibration whiteboard; (**5**) Sample table; (**6**) Dome uniform light source; (**Camera 1**) Visible near-infrared camera; (**Camera 2**) Short wave near-infrared camera.

The reflection images of eggs were collected using the reflection hyperspectral imaging system (Figure 1). The light source of this system is a dome uniform light source with a wavelength range of 50–2500 nm. The light source uniformly irradiates the egg on the electronically controlled moving platform. The reflected light of the egg is captured by the hyperspectral camera through the lens to obtain one-dimensional images and spectra. When the platform drives the egg to run continuously, continuous one-dimensional images and real-time spectra can be obtained. Note that the spectra are automatically recorded by the computer software. Finally, we obtained a three-dimensional data cube containing reflection image and spectral information.

**Figure 2:**
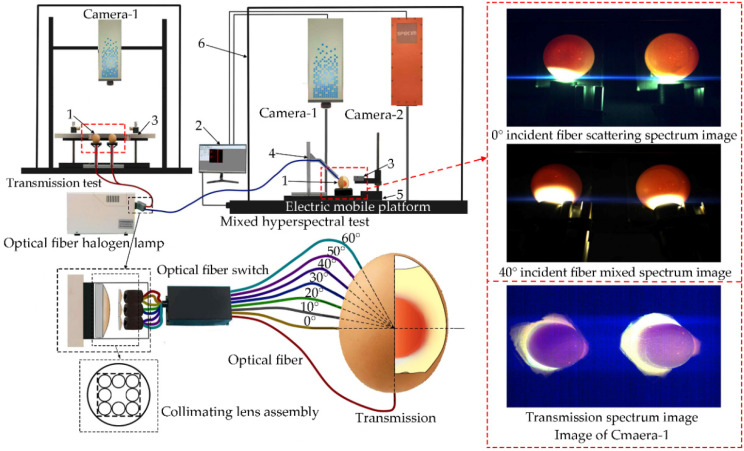
Optical fiber hyperspectral imaging system. (**1**) Pink-shell egg; (**2**) Computer; (**3**) Calibration whiteboard; (**4**) Optical fiber fixed metal frame; (**5**) Sample table; (**6**) Black box; (**Camera 1**) Visible near-infrared camera; (**Camera 2**) Short wave near-infrared camera.

The scattering, transmission, and mixed hyperspectral images of eggs were collected using an optical fiber hyperspectral imaging system (Figure 2). The light source of the system was an optical fiber halogen lamp (LG-150B, wavelength range 400–2500 nm). The incident angle of the fiber could be adjusted to collect the corresponding types of hyperspectral images. The scattering hyperspectral images were collected as the incident angle was 0°. The mixed hyperspectral images were collected as the incident angles were 10°, 20°, 30°, 40°, 50°, and 60°, respectively. The transmission hyperspectral images were collected as the light of fiber was shot directly under the egg. As the angle was selected, the platform drove the sample to move continuously to obtain continuous one-dimensional images and real-time spectral information. Finally, we obtained a three-dimensional data cube including scattering, transmission, and mixed images and spectral information.

### 2.3. Data Acquisition and Correction

The equipment was prepared before testing. The detection system was warmed up for 30 min. The height of “Camera 1” was set to 10 cm, the exposure time was 7 ms by adjustment and comparison. The height of “Camera 2” was set to 25 cm, and the exposure time was 9 ms. The conveyor belt speed was 0.36 cm/sec. The hyperspectral images were collected as follows: Ten eggs were randomly selected from the data group every day, and the larger end of the eggs (with air chamber) was placed upward under the dome uniform light source to obtain the reflection hyperspectral images. Then, they were placed in the transmission light and the optical fiber light sources with incident angles of 0°, 10°, 20°, 30°, 40°, 50°, and 60° to obtain the scattering, transmission, and mixed hyperspectral of the eggs. The tests were repeated and lasted for 28 days. The collected hyperspectral images were corrected in black and white because of the influence of dark current or uneven illumination on the experiment [21]. It was corrected by using SpecVIEW software established in the system and Equation (1) as follows:

(1) $$R=\frac{I_{0}-I_{b}}{I_{w}-I_{b}}$$

where R is the corrected spectral image, $I_{0}$ is the original spectral image, $I_{w}$ is the total reflection image of polyfluortetraethylene plate, $I_{b}$ is the all-black image by coving the lens.

### 2.4. Automatic ROI Extraction

Step 1 ROI mask

The images (R, 650; G, 550; B, 450) were exported by the software ENVI 4.8. The images were extracted by using MATLAB. They were binarized, and then operated by threshold segmentation, expansion, and erosion. Subsequently, their centroids were extracted and marked. We used threshold segmentation to extract regions with the color similar to eggshells. The regions contained only a small amount of glare. According to the selected area, the maximum horizontal and vertical lengths were calculated as the long axis and short axis of the ellipse. Finally, the center of the ellipse was used as the center of the original image, and the parameters of the long axis and the short axis were combined to fit and expand the ellipse image. Then, we extracted the ROI (region-of-interest) mask by the selected ellipse.

Step 2 Automatically extract the ROI of spectra

**Figure 3:**
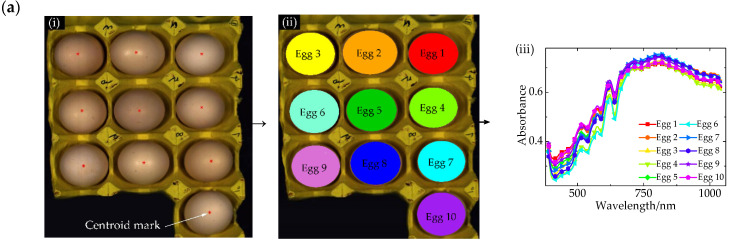

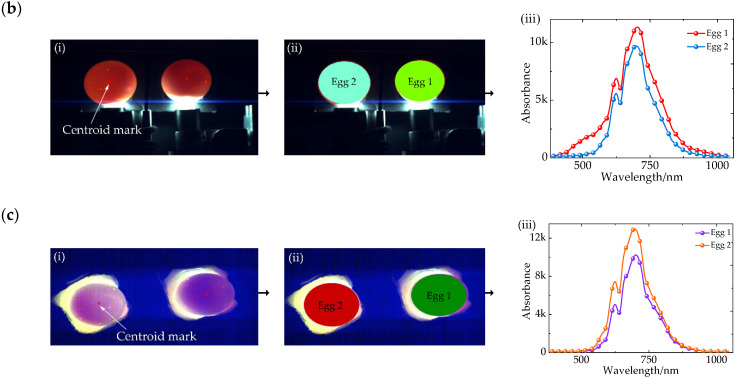
Region-of-interest (ROI) extraction processes. (**a**) Reflection; (**b**) Transmission; (**c**) Mixed hyperspectra; (**i**) Centroid mark; (**ii**) ROI; (**iii**) Original hyperspectral.

The positions of eggs in the mask image were extracted using the cell counting algorithm. The corresponding ROIs of eggs were determined and numbered by the settings of their mask images. These images were imported into ENVI. The average spectrum of a single ROI was used as the spectrum of an egg. The detailed processes are shown in Figure 3.

### 2.5. Determination of Haugh Unit

Five eggs were randomly selected from the calibration group every day, and they were numbered and weighed. For each egg, the shell were broken gently, and the height of protein was measured at 3 different points of 1 cm from the edge of their yolks. Three points were selected as far as possible, and the average height was used as the protein height of an egg. The Haugh units of the 5 eggs were calculated by Equation (2), and their average value was used as the egg freshness of the day [22] as follows:

(2) $$HU=100\times lg(h+7.57-1.7*w^{0.37})$$

where HU is Haugh unit of an egg, h (mm) is the average protein height of the three points; w (g) is the weight of an egg.

### 2.6. Spectrum Processing Method

It was necessary to preprocess the original spectra due to the uneven intensity of light sources at different wavelengths and the influence of instrument noise. In this paper, the spectra were processed using ten preprocessing methods, including multiplicative scatter correction (MSC) [23], standardized normal variate (SNV) [24], normalization [25], autoscales [26], mean centering (MC) [27], moving average method (MA) [28], detrend fluctuation analysis (Detrend) [29], Savitsky–Golay smoothing (SG) [30], Savitsky–Golay first derivative (SG-FD) [31], and Savitsky–Golay second derivative (SG-SD) [32]. To reduce calculation and increase calculation speed, competitive adaptive reweighted sampling (CARS) [33], principal components analysis (PCA) [34], and successive projections algorithm (SPA) [35] are preferable to extract feature wavelengths to reduce the dimensionality. The preprocessed dataset was used to extract feature wavelengths and used as the final sample. Then, 71.43% of the samples were randomly selected as the training set, and the remaining 28.57% 0% as the test set. We compared the prediction of egg freshness using the following six models: support vector machine (SVM) [36], k-nearest neighbor (KNN) [37], random forest (RF) [38], Naive Bayes (NB) [39], discriminant analysis classifier (DAC) [40], and latent Dirichlet allocation (LDA) [41]. In order to further improve the accuracy and the generalization ability of the egg freshness classification model, multiple weak classifiers were merged into a strong classifier by stacking ensemble learning [42].

## 3. Guided Filtering

### 3.1. Determination of Egg Haugh Unit

**Figure 4:**
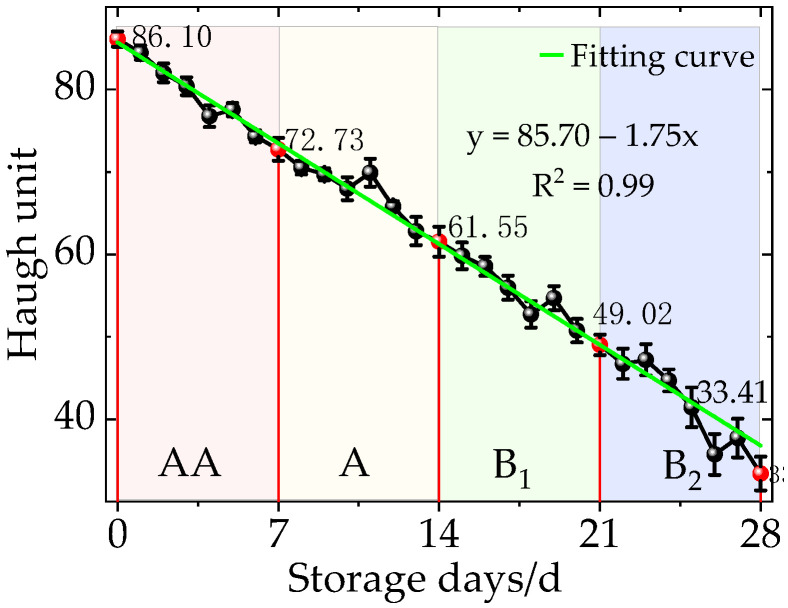
Haugh units of eggs versus time.

Five eggs were selected randomly every day to measure their Haugh units, and the units of 140 eggs were measured within 28 days. The units decrease linearly with time (Figure 4), and they fit well with Equation (3). Their detailed distribution is shown in Table 1.

(3) y=85.70−1.75x

It shows that the Haugh units range from 33.4 to 84.5, thus these eggs are edible. Their units are 84.5–72, 70.5–61.5, 59.8–49.0, and 47.2–33.4 in the first, second, third, and fourth weeks, and their freshness are classified as Grade AA, A, B_1_, and B_2_, respectively. After the fourth week, their units are below 30 and classified as Grade C, because their Haugh units gradually decrease. These eggs are easy to distinguish due to their obvious spoilage and unpleasant smell deterioration, therefore, they will not be discussed in this article.

### 3.2. Spectral Preprocessing

**Figure 5:**
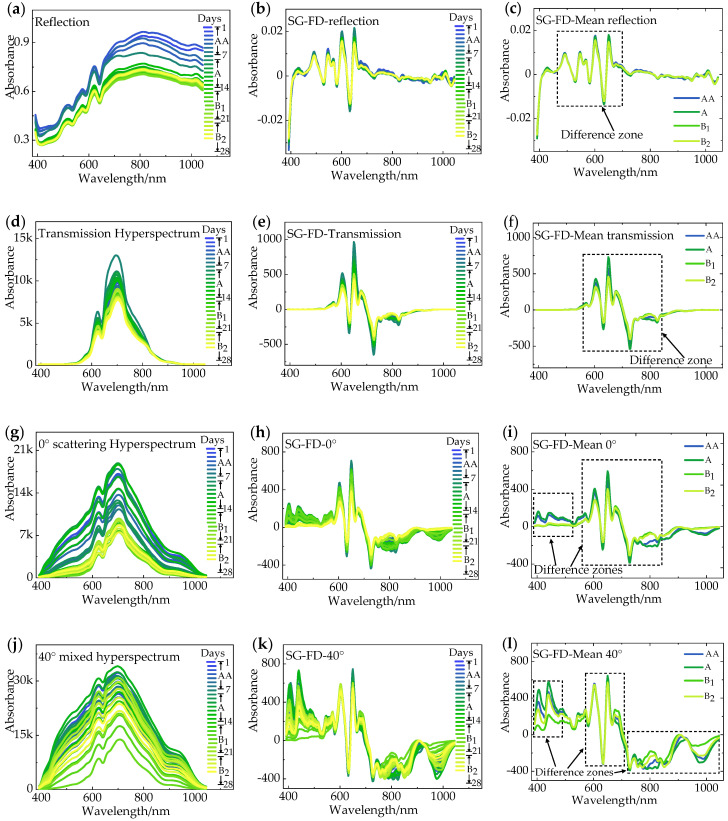
Savitsky–Golay first derivative (SG-FD) pretreatment. (**a**,**d**,**g**,**j**) The average hyperspectral of eggs per day from 1 to 28 days as incident light are reflection, transmission, 0° scattering, and 40° mixed spectra, respectively; (**b**,**e**,**h**,**k**) Corresponding spectra after SG-FD treatment; (**c**,**f**,**i**,**l**) The average spectra of Grade AA, A, B_1_, and B_2_ after SG-FD treatment, respectively.

The original spectra contain a lot of information about the freshness of an egg, however, it is impossible to find the law directly (Figure 5a,d,g,j). The spectra have obvious noise, which interferes with the later extraction of feature wavelength and modeling, and therefore reduces the accuracy of the prediction model. Therefore, the original spectra should be preprocessed separately. SG is an algorithm of polynomial smoothing and weighted average of moving windows based on the principle of least squares, whereas the main idea of FD is to obtain the first derivative of the spectrum, thereby amplifying the differences among different spectra. Herein, the original spectra are treated using SG-FD (Figure 5b,e,h,k). We obtain the average of the four Grade AA, A, B_1_, and B_2_, after the SG-FD treatment (Figure 5c,f,I,l). The obvious difference in spectra are mainly distributed in the 400–600, 550–800, 550–800, and 400–1000 nm in uniform reflection, transmission, 0° scattering, and 40° mixed spectra, respectively. This indicates that the difference in light source result in the egg information differences detected by hyperspectral images.

### 3.3. Feature Wavelength Extraction and Model Establishment

In our experiment, we use PCA, CARS, and SPA to extract the feature wavelengths and reduce the redundancy of the full-band original spectra, which eliminates irrelevant information, optimizes effective information, and establishes low-dimensional data models. Finally, different classification models are established according to the feature wavelength, and the best model is obtained by comparative analysis.

#### 3.3.1. Model Based on PCA

**Figure 6:**
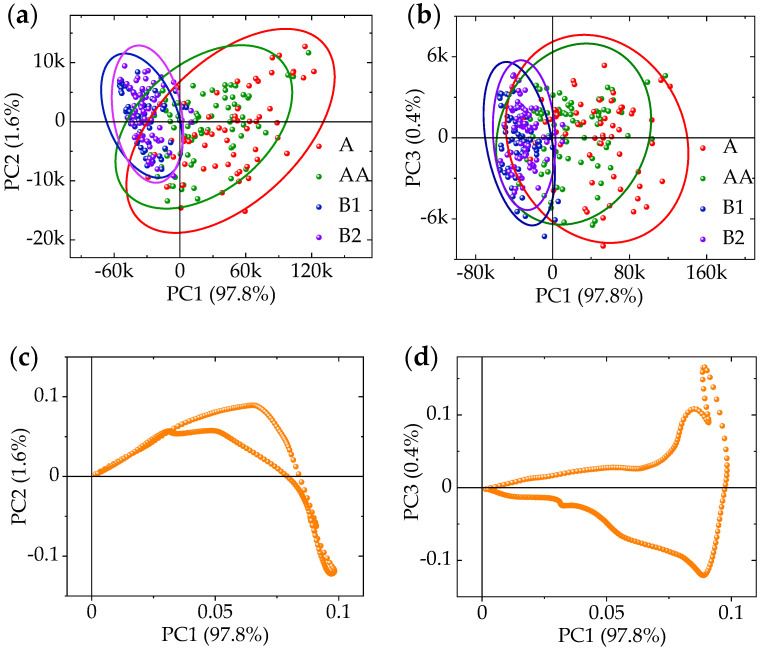
Principal components analysis (PCA) of 0° fiber light source based on Savitsky–Golay smoothing (SG) preprocessing. (**a**) Score plot of PC1-PC2; (**b**) Score plot of PC1-PC3; (**c**) Loading plot of PC1-PC2; (**d**) Loading plot of PC1-PC3.

The PCA analysis was based on preprocessed data. We performed a certain standardization and MC preprocess on the data before PCA. Figure 6a–d shows the PCA analysis results (PC1-PC2, PC1-PC3) in the case of 0° fiber light source based on SG preprocessing.

The contribution rate of the first, second, and third principal component are 97.8%, 1.6%, and 0.4%, respectively. The total contribution of these three components contains 99.79% of the spectral information, indicating that the feature wavelength can be reliably decomposed from the three components. Figure 6a,b shows the score plot of the AA, A, B_1_, and B_2_ levels of eggs, indicating that the A and AA levels of eggs are easily separated from the B_1_ and B_2_ levels in the principal component space. However, there are large overlaps between A and AA levels, B_1_ and B_2_ levels, and therefore it is difficult to distinguish them, i.e., we can easily distinguish the freshness and staleness of eggs based on PCA, but it is difficult to distinguish the more detailed level of freshness. Therefore, the accuracy of PCA may not be suitable for subsequent modeling. Figure 6c,d is the loading plot of the PCA model, which explains the contribution of each spectral value to the model establishment. The greater the coefficient of the spectral value, the greater the contribution rate to the model. The feature wavelength can be decomposed by searching the values with large coefficients to reduce the data dimension.

**Figure 7:**
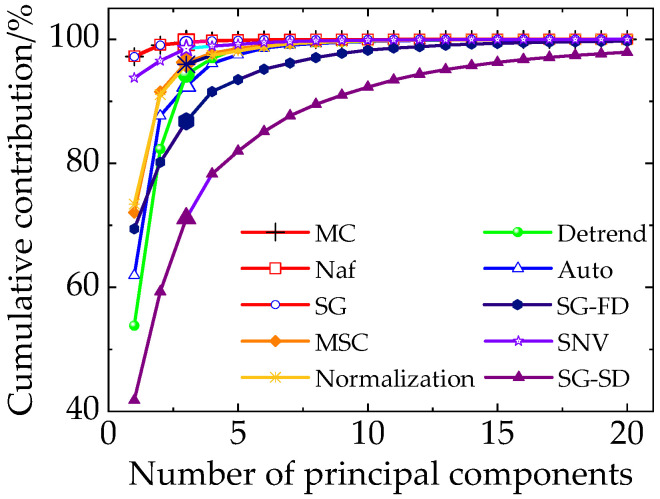
Cumulative contribution rates of the top 20 principal components in 0° incident light.

We take the various preprocessing methods of scattering hyperspectral at the 0° incident light as an example, calculate the cumulative contribution of the first 20 principal components (Figure 7). It shows that the first three principal components have the highest contribution. They were selected as feature component to extract the feature wavelengths.

Meanwhile, the cumulative contribution rates of different pretreatments are shown in Table 2. It can be seen that the cumulative contribution of the first three components for normalization, MC, MA, detrend, and SG are above 90%, which indicates that the feature wavelength can be reliably decomposed from the three components. Therefore, the first three components of these pretreatments are selected as the new coordinate system to reduce the dimension of the original spectra and extract the feature wavelengths.

Then, we establish LIBSVM, DCA, LDA, KNN, RF, and NB models to calculate the accuracy of training set and test set, respectively (Table 3). The results show that the overall accuracy is not high by using the weak classifier based on PCA. Among them, the classification accuracy of KNN and NB modeling is only 83.75%. The pretreatment of SG and MC have the best accuracy.

#### 3.3.2. Model Based on Successive Projections Algorithm (SPA)

**Figure 8:**
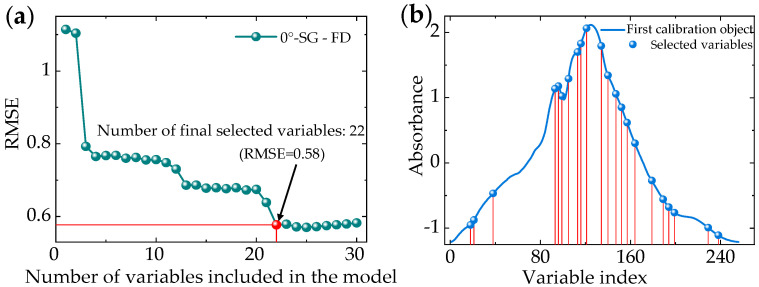
Number of variables in the 0° incident light model. (**a**) RMSE polyline; (**b**) Selected variables.

The successive projections algorithm (SPA) can eliminate collinear redundancy to find the wavelength segment with the minimum collinear information and represent the maximum information of the sample. In this experiment, the number of wavelengths selected by SPA was set to range from 5 to 30, and the step length was 1. Then, we iterated the data and selected the wavelength with the largest projection phasor as the feature wavelength combination. Meanwhile, the RMSE of different combinations was calculated by linear regression until the feature wavelength combination corresponding to the minimum RMSE was obtained. The SPA feature wavelength was extracted from the preprocessed data of SG-FD as the incident angle of 0°. The results show that the best RMSE = 0.58 as the feature wavelength is 22 (Figure 8).

The number of feature wavelength was extracted differently using different preprocessing methods (Table 4). Subsequently, LIBSVM, DCA, LDA, KNN, RF, and NB models were established to obtain the accuracy of the training set and the test set (Table 5).

By comparing Table 3 and Table 5, it can be concluded that the accuracy of feature wavelength extraction based on SPA is generally higher than that of PCA. In addition, the pretreatment of MSC, SNV, auto, and MC classified using the DAC model has higher accuracy; the 0° incidence angle MSC-SPA-DAC has the highest accuracy of 96.25%, while that of reflection incidence SG-SPA-LDA is 81.25%. These results are consistent in that the light of scattering has more internal information of an egg than that of reflection.

#### 3.3.3. Model Based on Competitive Adaptive Reweighted Sampling (CARS)

**Figure 9:**
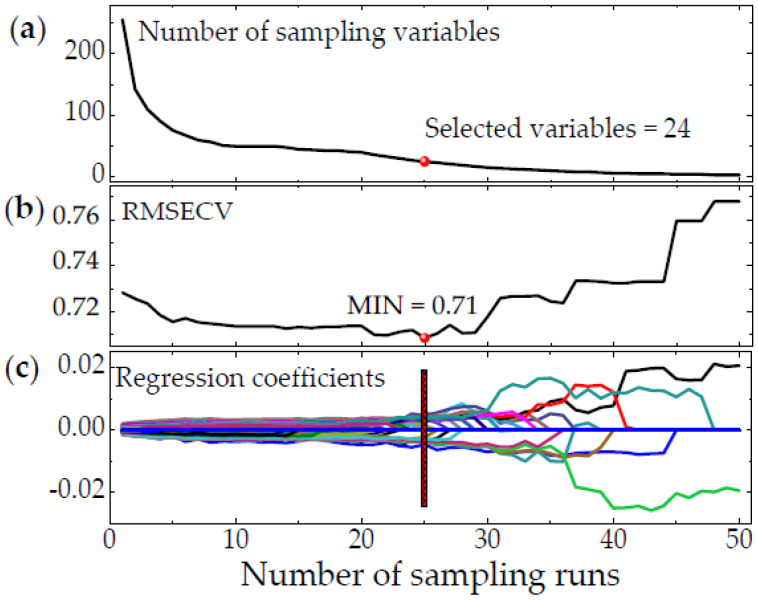
The extraction process of feature wavelength based on competitive adaptive reweighted sampling (CARS) at 0° incident source. (**a**) Number of sampling variables; (**b**) RMSECV; (**c**) Regression coefficient path.

Competitive adaptive reweighted sampling (CARS) is based on the principle of “survival of the fittest” in Darwin’s theory of evolution. In order to reduce the dimensionality, partial least squares are used to select the spectral value with a larger regression coefficient, and the value with a smaller one is eliminated to select some feature wavelengths for representing the full spectral information. After this preprocessing, the dimensionality of the data is effectively reduced. In this study, we reduced the dimensionality of the preprocessed spectrum by CARS and sampled the eggs by using Monte Carlo. The sampling time of Monte Carlo was set to 100, and the PLS model was established by using five-fold cross validation. Subsequently, the 0° incident light was taken as an example to extract the process of the feature wavelengths after SG-FD preprocessing (Figure 9).

The number of retained wavelengths decreases slowly after starting to decrease rapidly as the sampling frequency increases. RMSECV decreases slowly as the number of sampling runs ranges from 0 to 24, indicating that the eliminated wavelength has little influence on RMSECV. However, it increases significantly as the number exceeds 24, indicating that the feature wavelengths have been deleted. Therefore, the number of extracted feature wavelengths is 24. Similarly, the number preprocessed by other methods can be extracted (Table 6). Subsequently, the egg freshness classification models are established by LIBSVM, DCA, LDA, KNN, RF, and NB (Table 7).

It can be seen that the accuracy of weak classifier modeling based on CARS feature wavelength extraction is generally higher than that of SPA and PCA for egg freshness. The CARS classifier has a large number of models with high accuracies. Among them, DAC and KNN models have the highest accuracies. The models of 0° incident light SNV/Auto/SG-CARS-DAC, 0° incident light SNV-CARS-KNN, 40° incident light MSC-CARS-DAC/KNN, and 40° incident light detrend-CARS-RF have the highest accuracies of 95%. These indicate that the model corresponding to the 0° fiber light source has the highest accuracy and that of 40° fiber light source has the higher accuracy. Meanwhile, the model with uniform reflection light source has the lowest accuracy.

### 3.4. Best Prediction Model of Egg Freshness

The method presented in Section 3.3 is used for the nine different incident light modes to select their highest accuracy of egg freshness, respectively (Table 8). It shows that the overall model accuracy is extracted by the feature wavelength of CARS, which is higher than PCA and SPA. Among them, the weak classifiers DAC, KNN, and PCA have the three highest accuracies. Moreover, the accuracy of the MSC-SPA-DAC model (96.25) is the highest as the incident light angle is 0°. The accuracies of the 30° incident light using MA-CARS-KNN model and the 40° incident light using MSC-CARS-DAC model are 95% and 95%, respectively. These models with mean reflection light and 60° incident light have low accuracy, 86.25% and 87.5%, respectively. This indicates that the accuracy of the scattering hyperspectral model is higher than the other three models. In addition, as the angle of incidence increases, the overall accuracy decreases.

### 3.5. Egg Freshness Classification Based on Stacking Ensemble Learning

**Figure 10:**
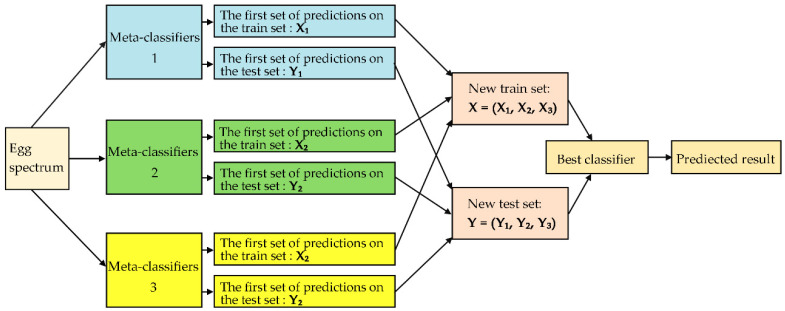
General flowchart for stacking ensemble learning.

To further improve the accuracy of the model, several weak classifiers are combined into a strong classifier, and stacking ensemble learning (SEL) [32] is performed to improve the generalization ability of the classification model. A two-layer training structure of SEL is used to improve the accuracy and speed of model. The overall flow chart of stacking ensemble learning is shown in Figure 10. The first layer uses different classifiers to establish different meta-classifiers and integrates the prediction results of all meta-classifiers. Then, the integrated data set of the classifiers with high accuracy in the first layer is used as the input of the second layer. Finally, the second layer is trained with the best classifier.

**Figure 11:**
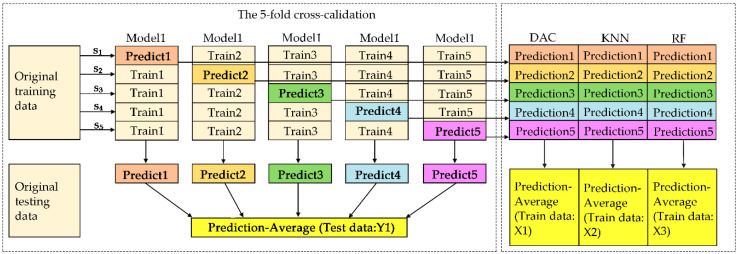
Training and prediction models for metamodels.

Therefore, in this experiment, three classifiers with the best model accuracy are selected to establish three meta-classifiers as the input of the second layer. The training and test set are predicted based on the idea of five-fold cross validation in each meta-classifier, in order to prevent data leakage (Figure 11). Finally, the new training and test set are used to establish the egg freshness classification model based on SEL.

The three classifiers, DAC, KNN, and RF, with the best accuracy are selected as the first layer. Meanwhile, the DAC model with the highest accuracy is selected as the second layer. Table 9 shows the results of the uniform reflection light source and transmission, 0° and 40° incident light sources.

It can be seen that the model based on SPA and CARS for extracting feature wavelength can finally achieve a higher accuracy than that of PCA. We compare the accuracies of different incident light corresponding models and find that the model of 0° fiber incident light source has the highest accuracy. Specifically, the 0° incident light source based on MSC-SPA can be increased from 96.25% to 100% (Table 10). That of the 40° fiber incident light source is higher. The accuracy of its SG/MSC-CARS-stacking and detrend-SPA-stacking models can reach 96.25%. While, that of uniformly reflected light source has the lowest accuracy, which is only 88.75% of its detrend-CARS-stacking model.

**Figure 12:**
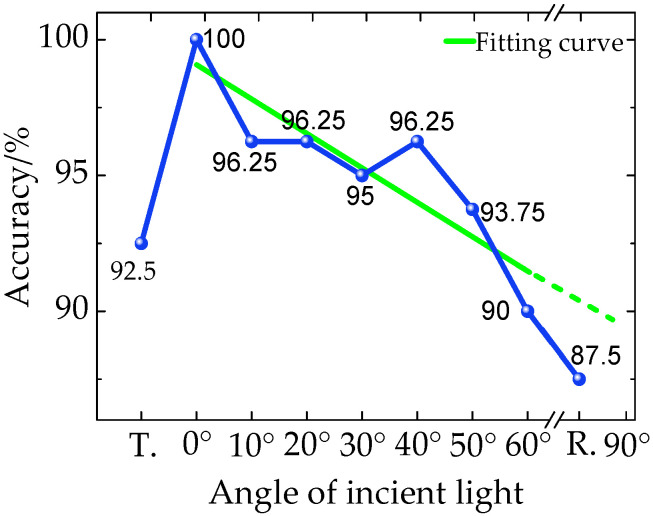
Classification accuracy-incident light line chart.

The highest accuracy of the best model is different under different incident angles (Figure 12). The accuracy at the 0° incident light (100%) is the highest. Its accuracy is almost linearly reduced from 100% to 90% as the incident angle increases from 0° to 60°. The accuracy of the transmission and reflection incident model are 92.5% and 87.5%, respectively. These indicate that the incident angle has an important influence on the accuracy of a model.

## 4. Discussion

The accuracy of the non-destructive detection model for egg freshness based on hyperspectral can be improved by using stacking ensemble learning. The learning is to use the output results of a series of models (base model) as the input features of the other models. This method realizes the stacking of models, that is, the outputs of the first layer model are used as the inputs of the second layer model. In operation, we need to pay attention to no leakage when combining the output of the first layer model. In addition, the data used for the output results of the basic model in the training samples cannot be used for training, in order to prevent overfitting of the final prediction. Note that validation on the training set is better than that on the test set. In order to prevent data leakage, it is necessary to output the results of each part of the sample separately by the k-fold method. In our experiment, we use the five-fold method (Figure 10) as follows: (1) We divide the data into five parts. One part at a time is used as the validation set, and the remaining four parts are used as the training set. In this way, a total of five models can be trained. (2) For the training set, one model is trained at a time to predict the validation set, and the prediction results are used as the second layer input of the corresponding samples in the validation set. The process is repeated five times, and obtain the outputs of each training sample that could be used as the input of the second layer model. (3) For the test set, one model is trained at a time to predict a result. Therefore, the sample in the final test set has five output results, and the average of these results is used as the input for the second layer. Therefore, in our experiment, the following six machine learning algorithms, LIBSVM, DCA, LDA, KNN, RF, and NB, are used to find the best combination of base-classifiers in the first stage and meta-classifier in the second stage. The three highest accurate classifiers, i.e., DAC, KNN, and RF, are used as the first layer. The training and test set are predicted based on the idea of five-fold cross validation in each metamodel to prevent data leakage (Figure 10). Finally, we obtain the first layer of data input into the second layer of the DAC model, and this method has the highest accuracy.

**Figure 13:**
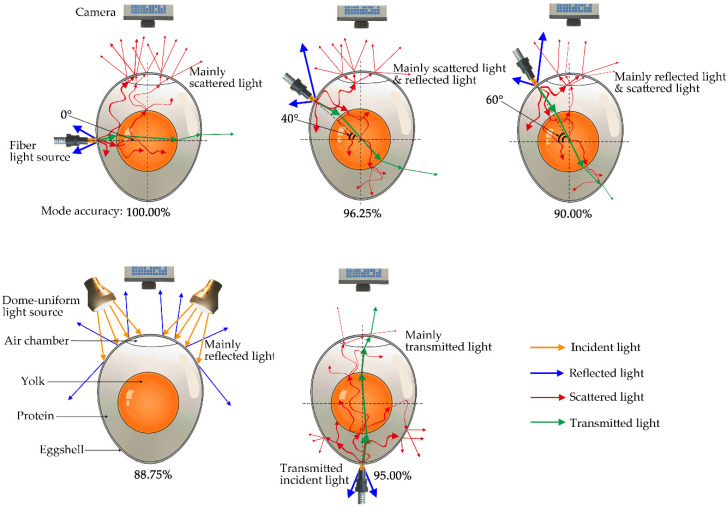
Light propagation inside an egg.

Different incident angles cause different information to be contained in the light collected by the camera, resulting in different accuracy of egg freshness. The freshness is closely related to the internal composition of an egg, yolk index [43], the pH of protein [1], and air chamber index [44]. The spectra collected about the more internal information of an egg is the precondition for establishing a model with higher accuracy. The analysis of the light propagation paths inside an egg helps us to understand the information contained in the image at different incident angles. For different incident modes, the propagation paths of light through an egg are different, and therefore the information collected is also different (Figure 13).

The camera mainly captures the reflected light of an egg as the incident light is a dome uniform light source, captures the scattered light through an egg as the incident angle is 0°, captures the reflection and the scattered light as the incident angle ranging from 0° and 60°, and captures the transmission light as the transmission fiber light. The scattered light through an egg carries out a lot of the biochemical information of the egg yolk, egg white, and air chamber. The reflected light by an egg only contains the information of the eggshell. The transmission light through an egg also carries out a lot of information, and the camera collects a higher proportion of the original light from the incident light source, resulting in a low accuracy. In our experiment, a camera captures a larger proportion of scattered light and a smaller proportion of reflection light as the incident angle is 0°, the accuracy of this angle is the highest. Meanwhile, the proportion of scattered light decreases and that of the reflection light increases as the incident angle increases gradually from 0° to 60°, causing the accuracy to decease gradually with an increase of the incident angle. The proportion of the reflection light should be the highest as the angle increases to 90°, thus its corresponding accuracy should be the lowest. In this mode, most of the light is reflected by the eggshell and captured by a camera. A small part of the light passes through the eggshell to enter the inside of the egg, but a larger proportion of the light shoots out from the bottom of the egg, which cannot be detected by the camera on the top of the egg. Therefore, only a very small part of the light is scattered on the upper of the egg and captured by the camera, resulting in its low accuracy. However, the 90° incident angle could not be tested due to the location conflict of the camera and the incident light source. However, the dome uniform light source is the light source with a weak intensity, which cannot nearly penetrate the eggshell, and only reflection light is captured by the camera. Thus, it is very similar to the 90° incident angle of the fiber light source. This is the reason why the accuracy of the model decreases linearly as the angle increases from 0° to 60° and R. (Table 10). For the transmitted light source, most of the light is reflected from the bottom of the egg, the scattered light from the lower layer of the egg is absorbed by the yolk, and only a small part of the scattered light from the upper layer is captured by the camera, and a large amount of the original light also interferes with the test accuracy. Hence, its detection accuracy is not high.

## 5. Conclusions

This paper has studied a method for improving the accuracy of egg freshness based on scattering hyperspectral, as well as researched the influence of different incident angles on the accuracy and explained its mechanism. The data processing process and conclusions are the following; (a) We established the classification model of egg freshness based on the combination of different preprocessing, feature wavelength extraction, and weak classifiers, and obtained the best classification models. We found that the 0° fiber light source MSC-SPA-DAC had the highest accuracy of 96.25%. Moreover, the detection accuracy of the 30° fiber light source MA-CARS-KNN and 40° fiber light source MSC-CARS-DAC were 95% and 95%, respectively. (b) Stacking ensemble learning was used to establish a fast egg freshness classification model to further improve the accuracy. In the 0° fiber optic light source MSC-SPA-stacking combination mode, the accuracy increased from 96.25% to 100%. (c) The hyperspectral classifier model of egg freshness was established under different incident light irradiation. Their highest accuracies of scattering, reflection, transmission, and mixed modes were 100.00%, 88.75, 95.00%, and 96.25%, respectively, indicating that the scattering hyperspectral for egg freshness detection was better than the other three. Moreover, the accuracy was inversely proportional to the incident angle, that is, the greater the incident angle, the lower the detection accuracy. Finally, this experiment realizes the non-destructive and high-precision detection of egg freshness based on scattering hyperspectral, and it has potential applications in online non-destructive detection.

## Figures and Tables

**Table 1:** Distribution of egg Hastelloy.

Freshness	Weeks	Max	Min	Average	Standard Deviation
AA	1	84.463	72.731	78.316	4.198
A	2	70.527	61.546	66.893	3.602
B_1_	3	59.824	49.019	54.493	3.962
B_2_	4	47.202	33.408	40.991	5.492

**Table 2:** Cumulative contribution rates of the first three principal components.

Pretreatment Method	Cumulative Contribution Rate/% (the First Three Principal Components)			
	Reflection	Transmission	0°	40°
MSC	89.16	94.97	96.31	95.64
SNV	89.01	96.84	92.50	88.63
Normalization	95.58	95.11	95.98	95.05
Auto	89.01	96.84	92.50	88.63
MC	99.31	90.70	99.51	99.15
MA	99.30	91.49	99.54	99.25
Detrend	91.63	98.99	94.38	90.89
SG	99.33	90.87	99.48	99.06
SG-FD	79.72	76.44	86.79	80.21
SG-SD	80.26	88.15	71.14	86.73

**Table 3:** The accuracy of the modeling based on PCA (the table shows the highest accuracy).

M.	P.	Training Set Prediction Accuracy/%				Prediction Set Training Accuracy/%			
		R.	T.	0°	40°	R.	T.	0°	40°
Libsvm	SG	60	89	51.5	51	47.5	81.25	47.5	52.5
DAC	MC	57.5	91.5	54.5	49	43.75	82.5	48.75	46.25
LDA	SG	72	67	91.25	83.5	63.75	65	81.25	60
KNN	SG	91.25	86.25	85	91.25	83.75	71.25	72.5	58.75
RF	MA	95	81.25	85	85	62.5	81.25	52.5	48.75
NB	SG	62.5	73.5	95	49.5	60	65	83.75	47.5

M., model; P., pretreatment; R., reflection; T., transmission; 0°, 0° incident light; 40°, 40° incident light.

**Table 4:** Number of feature wavelengths extracted after successive projections algorithm (SPA) processing.

P.	MSC	SNV	Norm.	Auto	MC	MA	Detrend	SG	SG-FD	SG-SD
R.	17	10	16	10	11	19	12	18	16	12
T.	20	18	17	18	12	15	21	20	12	6
0°	22	22	16	22	11	18	19	19	22	15
40°	35	48	47	26	24	16	26	35	35	44

N, number of feature wavelengths; P., pretreatment; R., reflection; T., transmission.

**Table 5:** The accuracy of the modeling based on SPA (the table shows the higher accuracies).

M.	P.	Training Set Prediction Accuracy/%				Prediction Set Training Accuracy/%			
		R.	T.	0°	40°	R.	T.	0°	40°
Libsvm	SG-FD	98.00	94.50	95.50	96.50	80.00	75.00	78.75	78.75
DAC	MSC	94.50	100.00	100.00	99.00	71.25	91.25	96.25	85.00
	SNV	90.00	100.00	100.00	100.00	68.75	90.00	93.75	92.50
	Norm.	87.00	99.50	99.00	99.50	62.50	90.00	91.25	91.25
	Auto	90.00	100.00	100.00	100.00	68.75	90.00	93.75	92.50
	MA	97.50	99.00	99.50	100.00	77.50	81.25	86.25	91.25
	Detrend	95.00	98.50	99.00	99.00	77.50	86.25	85.0	91.25
	SG	98.00	97.50	99.00	99.00	77.50	81.25	85.00	90.00
	SG-FD	97.00	97.50	98.00	98.50	77.50	77.50	92.50	90.00
LDA	SG	97.00	75.00	71.50	94.00	81.25	60.00	66.25	78.75
KNN	MSC	100	100	98.75	98.75	78.75	91.25	76.25	77.5
	Auto	100	100	100	98.75	86.25	91.25	91.25	87.5
	MC	98.75	98.75	100	100	76.25	87.5	76.25	91.25
RF	Detrend	94.50	100.00	100.00	99.00	71.25	91.25	96.25	85.00
	SG	90.00	100.00	100.00	100.00	68.75	90.00	93.75	92.5
	SG-FD	87.00	99.50	99.00	99.50	62.50	90.00	91.25	91.25
	SG-SD	90.00	100.00	100.00	100.00	68.75	90.00	93.75	92.50
NB	SNV	97.50	99.00	99.50	100.00	77.50	81.25	86.25	91.25
	Norm.	95.00	98.50	99.00	99.00	77.50	86.25	85.00	91.25
	MC	97.00	97.50	98.00	98.50	77.50	77.50	92.50	90.00

M., model; P., pretreatment; R., reflection; T., transmission; 0°, 0° incident light; 40°, 40° incident light.

**Table 6:** Number of feature wavelengths extracted after CARS processing.

P.	MSC	SNV	Norm.	Auto	MC	MA	Detrend	SG	SG-FD	SG-SD
R	16	43	26	32	32	14	46	32	40	46
T	24	35	29	24	20	28	23	27	24	36
0°	28	44	26	16	16	16	20	22	24	16
40°	16	24	16	24	16	21	19	20	19	15

N, number of feature wavelengths; P., pretreatment; R., reflection; T., transmission.

**Table 7:** The accuracy of the modeling based on CARS (the table shows the higher accuracies).

M.	P.	Training Set Prediction Accuracy/%				Prediction Set Training Accuracy/%			
		R.	T.	0°	40°	R.	T.	0°	40°
Libsvm	SG-SD	98.00	97.00	98.50	100.00	80.00	81.25	90.00	81.25
DAC	MSC	94.00	99.50	100.00	100.00	73.75	87.50	91.25	95.00
	SNV	98.00	100.00	100.00	100.00	81.25	90.00	95.00	90.00
	Norm.	97.50	100.00	100.00	100.00	80.00	92.50	93.75	88.75
	Auto	98.00	100.00	100.00	100.00	76.25	91.25	95.00	88.75
	MA	98.00	99.00	100.00	99.00	78.75	78.75	93.75	87.50
	Detrend	98.00	99.50	99.50	99.50	80.00	88.75	93.75	93.75
	SG	98.00	98.00	99.50	100.00	78.75	85.00	95.00	92.50
	SG-FD	98.00	99.00	100.00	99.50	82.50	78.75	92.50	92.50
	SG-SD	98.00	99.00	100.00	100.00	78.75	82.50	92.50	92.50
LDA	Detrend	100.00	78.50	85.00	92.50	86.25	66.25	58.75	78.75
KNN	MSC	100.00	100.00	100.00	100.00	73.75	87.50	91.25	95.00
	SNV	100.00	100.00	100.00	100.00	81.25	90.00	95.00	90.00
	Norm.	100.00	100.00	100.00	100.00	80.00	92.50	93.75	88.75
	Auto	100.00	100.00	100.00	100.00	76.25	91.25	95.00	88.75
	MC	100.00	100.00	100.00	100.00	80.00	80.00	86.25	90.00
	MA	100.00	100.00	100.00	100.00	78.75	78.75	93.75	87.50
RF	Detrend	94.00	99.50	100.00	100.00	73.75	87.50	91.25	95.00
	SG	98.00	100.00	100.00	100.00	81.25	90.00	95.00	90.00
	SG-FD	97.50	100.00	100.00	100.00	80.00	92.50	93.75	88.75
	SG-SD	98.00	100.00	100.00	100.00	76.25	91.25	95.00	88.75
NB	SNV	98.00	99.00	100.00	99.00	78.75	78.75	93.75	87.50
	Norm.	98.00	99.50	99.50	99.50	80.00	88.75	93.75	93.75
	Auto	98.00	98.00	99.50	100.00	78.75	85.00	95.00	92.50
	MC	98.00	99.00	100.00	99.50	82.50	78.75	92.50	92.50
	MA	98.00	99.00	100.00	100.00	78.75	82.50	92.50	92.50

M., model; P., pretreatment; R., reflection; T., transmission; 0°, 0° incident light; 40°, 40° incident light.

**Table 8:** The highest accuracy of a model under different incident lights.

Incident Light		The Best Model	Accuracy/%
Mean reflection light		Detrend-CARS-LDA	86.25
Optical fiber	Transmission	Nomalization-CARS-DAC	92.50
	0°	MSC-SPA-DAC	96.25
	10°	MA-CARS-PCA	93.75
	20°	SNV/Auto-SPA-DAC	92.50
	30°	MA-CARS-KNN	95.00
	40°	MSC-CARS-DAC	95.00
	50°	Detrend/SG-CARS-DAC, Detrend-SPA-DAC	91.25
	60°	SG-FD-SPA-KNN	87.50

**Table 9:** The accuracies of the stacking ensemble learning model (the table shows the highest accuracy).

M.	P.	Training Set Prediction Accuracy/%				Prediction Set Training Accuracy/%			
		R.	T.	0°	40°	R.	T.	0°	40°
PCA	SG-SD	100.00	97.50	100.00	97.50	81.25	86.25	82.50	82.50
SPA	MSC	94.00	99.50	100.00	100.00	75.00	93.75	100.00	90.00
	SNV	98.00	100.00	100.00	100.00	71.25	91.25	97.50	95.00
	Norm.	97.50	100.00	100.00	100.00	77.50	93.75	97.50	93.75
	Auto	98.00	100.00	100.00	100.00	72.50	93.75	95.00	95.00
	MA	98.00	99.00	100.00	99.00	78.75	83.75	88.75	95.00
	Detrend	100.00	99.50	99.50	99.50	82.50	92.50	88.75	96.25
	SG	98.00	98.00	99.50	100.00	85.00	83.75	91.25	92.50
	SG-FD	98.50	99.00	100.00	99.50	82.50	78.75	98.75	92.50
CARS	MSC	94.50	100.00	100.00	99.00	80.00	90.00	95.00	96.25
	SNV	90.00	100.00	100.00	100.00	87.50	92.50	97.50	92.50
	Norm.	94.50	99.50	99.00	99.50	85.00	95.00	95.00	92.50
	Auto	90.00	100.00	100.00	100.00	85.00	95.00	98.75	93.75
	MA	97.50	99.00	99.50	100.00	82.50	81.25	95.00	90.00
	Detrend	95.00	98.50	99.00	99.00	88.75	90.00	97.50	95.00
	SG	98.00	97.50	99.00	99.00	86.25	87.50	98.75	96.25
	SG-FD	98.00	97.50	98.00	98.50	86.25	83.75	98.75	95.00
	SG-SD	94.00	87.00	96.50	96.50	81.25	86.25	97.50	93.75

M., model; P., pretreatment; R., reflection; T., transmission; 0°, 0° incident light; 40°, 40° incident light.

**Table 10:** The highest accuracy of the best model under different incident modes.

Incident Light		The Best Model	Accuracy/%
Mean reflection light		Detrend-CARS	88.75
Optical fiber	transmission	Normalization/auto-CARS	95.00
	0°	MSC-SPA	100.00
	10°	SG-CARS	96.25
	20°	SNV-SPA, MSC-CARS	95.00
	30°	MA-CARS	96.25
	40°	SG/MSC-CARS, detrend-SPA	96.25
	50°	SG-CARS, MA/detrend-CARS	93.75
	60°	SG-FD-SPA	90.00
